# Fledgling Gaol Birds

**Published:** 1889-01-26

**Authors:** 


### BABIES.

*(Continued from p. 255.)*

### IV.—FLEDGLING GAOL BIRDS.

"I HAVE often thought," says Coleridge, "what a melancholy world this would be without children." There are several little worlds in London, teeming with population, alive with industries, worlds where men and women eat and drink, and sleep and work, and sing and pray, and sicken and die, and never a child toddles about in their midst; worlds where no child's prattle and laughter are ever heard. "What do you miss most in here?" was a question put the other day to a rough-looking fellow in one of the largest of the London prisons. "The young 'uns," said the man, without a moment's hesitation. He, of course, was speaking only for himself, but as one moved about among the crop-headed, drab-suited, melancholy-looking gang of convicts, working under the eyes of stalwart warders pacing to and fro with loaded carbines over their shoulders, one could not help wondering how many more of those hard-looking fellows were pining for the touch of little hands and the prattle of tiny tongues.

There are few perhaps who fully realise how much children are to them, or have been in one way or another, and there are few people more seriously to be pitied than many of the mothers in our prisons, when it becomes necessary to take their children from them at the threshold of a long term of penal servitude. Of course when a mother "gets into trouble," as the common expression goes, while she is nursing an infant, the child has to be taken into prison with her. Not infrequently, too, children are born in prison. Quite recently the writer of this article saw two little mortals who had just chanced on the dismal interior of Millbank as their starting point in life, and the mothers held them up for inspection at the gratings of their cells as proudly and smilingly as though they had been born in Buckingham Palace. They would be allowed to keep them till they were at least nine months old, by which time the weaning would be got over, or thereabouts, and the little creatures must then be handed over to the friends of the prisoners, if they have any friends who will take them, or to the workhouse if they have not. All infants are removable at nine months old, but under special circumstances mothers are permitted to retain them a little longer, and as a matter of fact they often keep them till they are twelve-months old. But at that age, if not before, all babies in prisons have to be parted from their mothers, and a very distressing parting it often is. Prisoners for the first nine months of their detention are always confined in separate cells. Each mother, therefore, is entirely shut up to the companionship of her baby, and for the first two or three months has nothing else to do but to attend to it. While her child is with her she never has more than a little light work—needlework, if she can do it; picking coir, if unable to do anything else—which the gaol doctor may limit or altogether dispense with, just as he pleases. It may easily be understood how completely wrapped up and absorbed in their children, under such circumstances, mothers will become as a rule. It is a rule not entirely without exception. A short time ago there was a woman in Millbank who seemed quite eager to get rid of her child—a bright, handsome little fellow, and she actually parted with him a month or two before she need have done, knowing that the little creature of nine or ten months old would go from her arms to the custody of strangers in the nearest workhouse. There is, of course, a good deal of difference in mothers who are in prison, as well as in those who are out of it; but, as a rule, this parting, after ten or twelve months of the closest companionship, and just as the little ones are beginning to grow playful and interesting in their ways, is a terrible wrench. Few persons of ordinary sensibility could have failed to be painfully impressed by the look of heart-breaking anguish that came into one of the hardest and most repulsive-looking female faces ever seen, even in a gaol, when the other day incidental reference was made to the fact that she would shortly have to part with the little one she held in her arms, and who evidently regarded the arrival of visitors as a rare opportunity for a little fun. It was distressing to see the eager clutching of the child to her breast, and the equally eager grasping at what seemingly she took to be some gleam of hope that her visitors might have the power to spare her the dreaded separation. She was doomed to five years' penal servitude.

The interesting building in Tothill Fields used to be the London women's prison. It was a peculiarly-constructed place; and they had one small block which they called the nursery, and in which were confined all the prisoners with children in arms. Everywhere else throughout the gloomy, great enclosure there was depressing silence. Order, discipline, repression, unbroken sadness and gloom prevailed everywhere, and everywhere the very air seemed heavy with guilt and crime. It was singular, indeed, to hear the customary rattle of huge keys, the throwing open of a heavy iron door, and then to be met with the crowing and prattle of two or three dozen little desperadoes, utterly defiant of all prison rules, and seemingly bent on living out the sentiment that—

> "Stone walls do not a prison make,
> Nor iron bars a cage."

At the present moment Millbank contains the female prisoners who would formerly have been sent to Tothill Fields, and here also they have a range of cells set apart as a nursery. It is odd to go along from one to another and find in every one of them a wondering little innocent, many of them as bright and pert and as pretty as though born in a palace, and quite as unconscious of their ignominy. They are certainly far better off than tens of thousands of babies in the slums of London. All prison children are, of course, kept scrupulously clean, and if the mothers are not able to suckle them the Home Office requires that an ample supply of good milk shall be provided, or whatever else the medical officer may think fit to prescribe. The diet for a child under three months is left entirely to the discretion of the doctor. From three to six months each little prisoner gets half a-pint of milk daily, and from six to nine months it is to have six ounces of bread and a pint of milk every day, and half a-pint of beef-tea three times a-week. The cells are comfortably warm, and they have good beds, and, as it has been said, mothers have nothing to do but to give them all the attention they require. Whenever the day is fine they take them out for an hour's exercise.

It is not surprising that prison babies thrive immensely, as a rule, compared with thousands outside the prisons, and many a little unfortunate who goes into gaol with its mother, a poor puny, wizened little mortal, enjoys a clean skin, comfortable quarters, proper nursing, and abundance of wholesome food for a few months, and goes out into the rough world again quite another being. Often the most indifferent mothers will become most devoted to their children in their new aspect. Passing along "the nursery" at Millbank, it is difficult to realise that many of the mothers who hold up their children for inspection at the grated doors of their cells are of the most drunken and abandoned characters, and that the pretty children that many of them hold up are the waifs of London slums. It is curious to see how proud and pleased almost all of them look as they hold up their cherubs to the dull light stealing down through their prison bars. The children, like their mothers, have a prison garb, and very pretty many of them look in it. It is a light blue check frock, with a blue check pinafore of a somewhat larger pattern; and many of the women will contrive to get a bit of something to tie up the sleeves at the shoulders. Really many of the little things look very winsome and pretty, and painful enough must be the parting from them. The stranger who goes from cell to cell full of sympathy and compassion, however, would perhaps do well very carefully to observe the prison rule, which forbids any conversation with prisoners, or must be prepared for the possibility of some rather sensational shocks. Here, for example, is a rather pretty, interesting-looking young woman, evidently very proud of the bright-eyed little creature she holds up to the bars. "How long are you in for?" asks the visitor, chucking the little one under the chin. Those mild, modest eyes instantly drop, and there is a quiver of the full passionate lip. "For life," is the dejected answer. Shocked, and half-incredulous, the visitor turns to the female warder, who strictly speaking ought not to make any revelations, but who presently whispers, "She murdered her first child; that one is the second." "We had a case the other day," said a prison official a short time time since, "really very distressing to witness. The child had to be removed, and it seemed like tearing the very heartstrings out of the mother." Human nature is indeed a strange compound. That woman so passionately attached to her child had had a previous one, and she was there undergoing five years' penal servitude because she had cut its throat and left it to die miserably in a field.

*(To be continued.)*

